# Neural-Based Compensation of Nonlinearities in an Airplane Longitudinal Model with Dynamic-Inversion Control

**DOI:** 10.1155/2017/8575703

**Published:** 2017-12-19

**Authors:** YanBin Liu, YuHui Li, FeiTeng Jin

**Affiliations:** ^1^Key Laboratory of Unmanned Aerial Vehicle Technology of Ministry of Industry and Information Technology, Nanjing University of Aeronautics and Astronautics, Nanjing 210016, China; ^2^College of Astronautics, Nanjing University of Aeronautics and Astronautics, Nanjing 210016, China

## Abstract

The inversion design approach is a very useful tool for the complex multiple-input-multiple-output nonlinear systems to implement the decoupling control goal, such as the airplane model and spacecraft model. In this work, the flight control law is proposed using the neural-based inversion design method associated with the nonlinear compensation for a general longitudinal model of the airplane. First, the nonlinear mathematic model is converted to the equivalent linear model based on the feedback linearization theory. Then, the flight control law integrated with this inversion model is developed to stabilize the nonlinear system and relieve the coupling effect. Afterwards, the inversion control combined with the neural network and nonlinear portion is presented to improve the transient performance and attenuate the uncertain effects on both external disturbances and model errors. Finally, the simulation results demonstrate the effectiveness of this controller.

## 1. Introduction

For a general longitudinal model of the airplane, the flight control law tends to be designed in terms of the linearized model corresponding to the given trim points. On this basis, the proportional-integral-derivative (PID) controller is used to achieve the desired flight performance under the assumption that the short-period dynamics are faster than the phugoid mode [1]. However, the classical PID controller may be limited due to too many parameters that need to be scheduled and optimized for the strong coupling airplane model under the complicated flight condition. As a result, the inversion design approach is a very useful tool in the control design [2], and the main advantage lies in avoiding the iterative regulation concerning the control parameters, and this controller provides greater flexibility for the strong coupling system [3]. More importantly, the control design using the dynamic-inversion method is based on the nonlinear model instead of the interpolated linear model [4].

In some studies, the inversion control design is realized by adopting feedback signals to offset inherent coupling dynamics, thus guaranteeing the satisfactory decoupling control ability. In particular, an investigation example was illustrated using the dynamic-inversion methodology for the linear model of a generic X-38 type reentry vehicle [5]. Correspondingly, the closed-loop stability and robustness of a dynamic-inversion flight controller for reentry vehicles were quantified in consideration of the influence along with the different flight dynamics. In addition, a methodology was presented using a combination of the linear dynamic-inversion controller and adaptive filter in order to implement MIMO reconfigurable flight control [6]. Such control design could improve significantly the tracking performance, handling qualities, and PIO tendencies for the closed system. Besides that, Doman and Ngo [7] discussed an indirect adaptive control problem by applying a baseline dynamic-inversion control structure. Furthermore, a quaternion-based attitude controller was developed based on the inversion control approach for the X-33 in the ascent flight phase. The dynamic-inversion control approaches were introduced for a spacecraft, not only an airplane, to realize the attitude control in response to the servo-constraint dynamics [8]. This control law consisted of particular and auxiliary parts wherein the particular part played a role in driving the spacecraft attitude variables, whereas the auxiliary potion provided the necessary internal stability with the aid of the involved null-control vector. In general, the inversion method is adopted in the control design for both the airplane and spacecraft models in recent years. It is noted that the main difference between the inversion approach and conventional method lies in that the resulting design model is achieved by the state feedback, thus keeping the exact dynamics in contrast to the approximating linearization [9].

In this paper, the flight control law is proposed using the neural-based inversion design method and nonlinear compensation for a general longitudinal model of the airplane. In particular, the dynamic-inversion control can relieve the strong coupling effects regarding the model dynamics, whereas the neural-based compensation is helpful in improving the robust performance to suppress the uncertain disturbances. There are three aspects of this problem that have to be addressed. First, the inversion design method is introduced to convert the nonlinear mathematic model to the equivalent model accurately. After that, the inversion control law is designed to stabilize the system and relieve the coupling effects. Furthermore, the compensation using the neural network and nonlinear portion is introduced to improve the transient performance and system robustness. Lastly, an airplane example is provided to verify the feasibility of the proposed controller.

## 2. Longitudinal Model of an Airplane

The longitudinal motion of the airplane involves only vertical motion parameters and aerodynamic actions, so the airplane dynamics can be described based on the velocity coordinate. While the elevator deflection (*δ*_*e*_) and throttle setting (*η*_*c*_) are selected as control inputs (*U*), the airplane model with the state variables *X* = [*V*, *γ*, *q*, *α*, *h*] is given as follows [10]:

(1) V˙=Tcos⁡α−Dm−gsin⁡γ,γ˙=L+Tsin⁡αmV−gcos⁡γV,q˙=MyIy,α˙=q−γ˙,h˙=Vsin⁡γ,

where *m* and *I*_*y*_ denote the mass and moment of inertia of the airplane, respectively. Besides that, the lift *L*, the drag *D*, the thrust *T*, and the pitching moment *M*_*y*_ are determined by

(2) L=12ρV2SwCL,D=12ρV2SwCD,My=12ρV2Swc−CM,T=12ρV2SwCT.

In (2), *S*_*w*_ and $\overset{-}{c}$ represent the reference area and mean aerodynamic chord, respectively. Furthermore, we assume that the lift coefficient *C*_*L*_, drag coefficient *C*_*D*_, pitching moment coefficient *C*_*M*_, and propulsive coefficient *C*_*T*_ in this work are approximately stated by

(3) CL=fLρ,V,α,CD=fDρ,V,α,CT=fTρ,V,α,ηc,CM=fMyρ,V,α,q+fδeδe.

Also, the gravity constant (*g*) and air density (*ρ*) as a function of altitude are shown by

(4) g=fgh,ρ=fρh.

Based on (1)–(3), the balance restrictions are provided by

(5) QfTfρhd,Vd,αr,ηcrcos⁡αr−QfDfρhd,Vd,αrm=0,QfLfρhd,Vd,αr+QfTfρhd,Vd,αr,ηcsin⁡αrmVr=fghdVd,Q=12fρhdVd2Sw,fMyfρhd,Vd,αr+fδeδer=0.

For any *V*_*d*_ and *h*_*d*_ in (5), the trim flight parameters regarding *α*_*r*_, *η*_*cr*_, and *δ*_*er*_ can be solved. To this end, the inversion control system, in accordance with whether accurate feedback linearization or Taylor linear approximation is used, can be designed based on these obtained trim values.

## 3. Inversion Control Laws Based on Accurate and Approximate Equivalent Model

For the nonlinear model of the airplane in (1), its inversion model can be derived by applying, respectively, the differential geometry theory and small perturbation theory. Correspondingly, the feedback linearization method transforms the nonlinear model of the aircraft to the equivalent model which keeps completely the high-order dynamics of the original model. As a result, not only is the resulting inversion model based on the feedback linearization more accurate than that based on the approximate linearization, but also the control capacity is enhanced in that the uncertain effects of the approximate linearization are removed accordingly [11].

### 3.1. Inversion Control Law Using Feedback Linearization

First, selecting *V* and *h* as the system outputs, the derivative of $\dot{V}$ corresponding to *Y* = [*V*, *γ*, *α*, *η*, *h*]^*T*^ is deduced based on the feedback linearization idea and differential geometry theory [12], and it is expressed by

(6) V¨=ω1Y˙m,ω1T=ω11ω12ω13ω14ω15=∂T∂Vcos⁡α−∂D∂V−mgcos⁡γ∂T∂αcos⁡α−Tsin⁡α−∂D∂α ∂T∂ηcos⁡α ∂T∂hcos⁡α−∂D∂h−m∂g∂hsin⁡γ,η¨=−Aη˙−Bη+Bηc,

where *η* is the intermediate variable that needs to be adopted, so, further differentiating $\ddot{V}$ with respect to *Y*, we have

(7) V3=ω1Y¨+Y˙TΩ1Y˙m,Ω1=dω1dY=dω11dY,dω12dY,dω13dY,dω14dY,dω15dY.

Considering the presence of $\ddot{Y}={\left[\ddot{V},\ddot{\gamma },\ddot{\alpha },\ddot{\eta },\ddot{h}\right]}^{T}$ in (7) and simultaneously combining it with

(8) α¨=q˙−γ¨=MyIy−γ¨=QfMyρ,V,α+QfδeδeIy−γ¨=QfMyρ,V,αIy−γ¨+QfδeIyδe=C+Dδe,

then we have

(9) V3=ω1Y¨+Y˙TΩ1Y˙m=ω1Y¨0+Y˙TΩ1Y˙m+ω13Dmδe+ω14Bmηc=V03+g11δe+g12ηc,Y¨0=V¨,γ¨,C,−Aη˙−Bη,h¨T.

Equation (9) shows that the expression of *V*^(3)^ includes the control inputs *δ*_*e*_ and *η*_*c*_, indicating that the nonlinear model has been partially transformed into the linear system [13]. Alternatively, higher order differential equations of *h* can be deduced as

(10) h¨=V˙sin⁡γ+Vγ˙cos⁡γ,h3=V¨sin⁡γ+2V˙γ˙cos⁡γ−Vγ˙2sin⁡γ+Vγ¨cos⁡γ,h4=V3sin⁡γ+3V¨γ˙cos⁡γ−3V˙γ˙2sin⁡γ+3V˙γ¨cos⁡γ−3Vγ˙γ¨sin⁡γ−Vγ˙3cos⁡γ+Vγ3cos⁡γ.

In (10), the second derivative of the flight path angle *γ* with regard to *Y* = [*V*, *γ*, *α*, *η*, *h*]^*T*^ is written as

(11) γ¨=π1Y˙,π1T=π11π12π13π14π15=∂L/∂V+∂T/∂Vsin⁡αmV−L+Tsin⁡αmV2+gcos⁡γV2gsin⁡γV∂L/∂α+∂T/∂αsin⁡α+Tcos⁡αmV∂T/∂ηsin⁡αmV∂L/∂h+∂T/∂hsin⁡αmV−∂g/∂hcos⁡γV.

Similarly, the differentiation of $\ddot{\gamma }$ regarding *Y* = [*V*, *γ*, *α*, *η*, *h*]^*T*^ is obtained by

(12) γ3=π1Y¨+Y˙TΩ1Y˙=π1Y¨0+Y˙TΩ1Y˙+π13Dδe+π14BηcΩ1=dπ1dY=dπ11dY,dπ12dY,dπ13dY,dπ14dY,dπ15dYT.

Substituting (9) and (12) into (10), we have

(13) h4=ω1Y¨0+Y˙TΩ1Y˙sin⁡γm+3V¨γ˙cos⁡γ−3V˙γ˙2sin⁡γ+3V˙γ¨cos⁡γ−3Vγ˙γ¨sin⁡γ−Vγ˙3cos⁡γ+Vcos⁡γπ1Y¨0+Y˙TΩ1Y˙+π13Dcos⁡γ+ω13Dmsin⁡γδe+π14Bcos⁡γ+ω14Bmsin⁡γηc=h04+g21δe+g22η.

With the integration of (9) and (13), we get

(14) $$\begin{array}{c} \left[\begin{array}{c} V^{\left(3\right)}\\ h^{\left(4\right)} \end{array}\right]=\left[\begin{array}{c} V_{0}^{\left(3\right)}\\ h_{0}^{\left(4\right)} \end{array}\right]+\left[\begin{array}{cc} g_{11} & g_{12}\\ g_{21} & g_{22} \end{array}\right]\left[\begin{array}{c} \delta_{e}\\ \eta_{c} \end{array}\right]=F_{0}+GU. \end{array}$$

If the matrix *G* is invertible, let

(15) $$\begin{array}{c} \left[\begin{array}{c} V^{\left(3\right)}\\ h^{\left(4\right)} \end{array}\right]=\left[\begin{array}{c} \nu_{1}\\ \nu_{2} \end{array}\right]=\nu , \end{array}$$

where *ν* represents the so-called pseudo-control vector [14], so the inversion model of the airplane is built by

(16) $$\begin{array}{c} U=G^{-1}\left(\;v-F_{0}\;\right)=f^{-1}\left(\;X,v\;\right). \end{array}$$

As long as the output of (16) is regarded as the input of the airplane model and at the same time (15) holds, the decoupling control goal can be achieved for the nonlinear airplane model. Furthermore, we define tracking errors as [14]

(17) SV=V¨−V¨d+2λVV˙−V˙d+λV2V−Vd,Sh=h3−hd3+3λhh¨−h¨d+3λh2h˙−h˙d+λh3h−hd,

where *V*_*d*_ and *h*_*d*_ represent command signals, respectively. Differentiating *S*_*V*_ and *S*_*h*_ and simultaneously combining them with (14), we have

(18) S˙VS˙h=RVRh+g11g12g21g22δeηcRV=2λVV¨−V¨d+λV2V˙−V˙d+V03−Vd3,Rh=3λhh3−hd3+3λh2h¨−h¨d+λh3h˙−h˙d+h04−hd4.

Let the inversion control law be [15]

(19) $$\begin{array}{c} \left[\begin{array}{c} \delta_{e}\\ \eta_{c} \end{array}\right]={\left[\;\begin{array}{cc} g_{11} & g_{12}\\ g_{21} & g_{22} \end{array}\;\right]}^{-1}\left[\begin{array}{cc} -R_{V} & -k_{V}S_{V}\\ -R_{h} & -k_{h}S_{h} \end{array}\right] \end{array}$$

so we have

(20) SVS˙V=−kVSV2<0,ShS˙h=−khSh2<0.

In (20), *S*_*V*_ and *S*_*h*_ will converge to zero exponentially by choosing *k*_*V*_ and *k*_*h*_ as properly positive constants, while making track errors *V* − *V*_*d*_ and *h* − *h*_*d*_ reach zero rapidly [15]. Furthermore, the measurement errors in relation to the system outputs and state variables are considered in (15) and (16), and we have

(21) $$\begin{array}{c} U={\left(\;G+\Delta G\;\right)}^{-1}\left[\;\left(\;v+\Delta v\;\right)-\left(\;F_{0}+\Delta F_{0}\;\right)\;\right], \end{array}$$

where Δ*G*, Δ*v*, and Δ*F*_0_ are the uncertainties caused by the sensor errors. Accordingly, tracking errors in (17) change to *S*_*V*_ + Δ*S*_*V*_ and *S*_*h*_ + Δ*S*_*h*_. In this case, if the control law in (19) is selected, the Lyapunov stability in (20) may not be satisfied. Therefore, it is necessary to apply the adaptive signals to offset the uncertain effect in relation to the sensor noise as a result of ensuring the global stability throughout the overall flight envelope.

### 3.2. Inversion Control Law Using Approximate Linearization

The approximate linearization approach is considered that the airplane movement is associated with small deviations from the steady flight state. And all high-order dynamics are regarded to be small such that their actions are negligible in contrast to the first-order model dynamics. When the first-order terms are kept in (1) and (5) using the approximate linearization method [16], then the following linear equations are obtained:

(22) ΔX˙=ΔV˙Δγ˙Δq˙Δα˙Δh˙=−XV−g0−Xα0ZV00Zα0−MV0Mq−Mα0−ZV01−Zα00Vr000ΔVΔγΔqΔαΔh+0Xηc−Zδe0MδeMηcZδe000ΔδeΔηc=AΔΔX+BΔΔU.

Correspondingly, the inversion control law based on this approximate model is expressed by

(23) $$\begin{array}{c} \left[\begin{array}{c} \delta_{e}\\ \eta_{c} \end{array}\right]=\left[\begin{array}{c} \delta_{er}\\ \eta_{cr} \end{array}\right]+pinv\left(\;B_{\Delta }\;\right)\left(\;\Delta v-A_{\Delta }\Delta X\;\right), \end{array}$$

where pinv represents the pseudo-inverse function, and let

(24) Δv=kVpV−Vc+kVdV˙kγθkqqkαθkhph−hc+khdh˙=ΔX˙.

In (24), if control parameters are selected suitably, Δ*v* will approach $\Delta \dot{X}$ such that the inversion control based on the approximate linearization principle can be realized in the given flight condition.

## 4. Robust Adaptive Control with Neural-Based Compensation of Nonlinearities

Improving the transient performance is very important for the aircraft model to follow the expected command rapidly without deviating from the design point. Alternatively, the system robustness will guarantee flight stability with the existence of the large model uncertainties and external disturbances. As a result, the transient performance and system robustness can be an issue for the aircraft model to realize the challenging tasks.

To this end, this work combines the above dynamic-inversion control with the compensation of the neural network and nonlinear potion in order to ensure system robustness and self-adaption and to improve the transient performance. This is because the inversion control is sensitive to modeling errors due to the need of the detailed knowledge of the nonlinear airplane model. In this case, the application of the neural network can alleviate this sensitivity, and the nonlinear portion can ameliorate the transient performance associated with the inversion controller [17].

First, the inversion design idea based on the feedback linearization principle transforms the nonlinear model in (1) to a standard form in (14). Correspondingly, the inverse model with the uncertain parts is expressed by

(25) $$\begin{array}{c} U={\hat{G}}^{-1}\left(\;\hat{v}-{\hat{F}}_{0}\;\right)={\hat{f}}^{-1}\left(\;X,\hat{v}\;\right). \end{array}$$

Afterwards, the inversion error is defined by

(26) $$\begin{array}{c} \varepsilon =f\left(\;X,U\;\right)-\hat{f}\left(\;X,U\;\right). \end{array}$$

Based on (25) and (26), (15) is rewritten as

(27) $$\begin{array}{c} \left[\begin{array}{c} V^{\left(3\right)}\\ h^{\left(4\right)} \end{array}\right]=\left[\begin{array}{c} {\hat{\nu }}_{V}\\ {\hat{\nu }}_{h} \end{array}\right]=\left[\begin{array}{c} \nu_{V}+\varepsilon_{V}\\ \nu_{h}+\varepsilon_{h} \end{array}\right]=\nu +\varepsilon . \end{array}$$

Furthermore, the pseudo-control vector consisting of the proportional controller, command derivative, and adaptive signal is selected [18], and it is expressed as

(28) νV=vVp+Vd3−v^adV,νh=vhp+hd4−v^adh,

where

(29) vVp=kVpVd−V+kVd1V˙d−V˙+kVd2V¨d−V¨,vhp=khphd−h+khd1h˙d−h˙+khd2h¨d−h¨+khd3hd3−h3.

After substituting (28) and (29) into (27), we have

(30) eV3=−kVpeV−kVd1e˙V−kVd2e¨V+v^adV−εV,eh4=−khpeh−khd1e˙h−khd2e¨h−khd3eh3+v^adh−εh.

By selecting the suitable control parameters, (30) can become Hurwitz such that the zeros of the resulting polynomial are all in the left half of the complex plane [19]. Not only that, but also the feasible selection of ${\hat{v}}_{adV}$ and ${\hat{v}}_{adh}$ can ensure that the low damping ratio is provided to achieve fast rising and regulating time when the tracking error is large. In turn, the higher damping ratio is given to decrease the overshoot when the output reaches the anticipated target. More importantly, ${\hat{v}}_{adV}$ and ${\hat{v}}_{adh}$ can further cancel the effects of uncertain errors as a result of the fact that*e*_*V*_ and *e*_*h*_ can approach zero and the control goal corresponding to the adaptive command track can be realized [20].

To this end, the adaptive compensation includes the nonlinear portion and output of the neural network, and it is provided as

(31) v^adV=−ρVEVTPVBV+w^pVTξpV,v^adh=−ρhEhTPhBh+w^phTξph,ξpV=ξph=exp−πσ2X−ϖ2,

where $E_{V}={\left[e_{V},{\dot{e}}_{V},{\ddot{e}}_{V}\right]}^{T}$, $E_{h}={\left[e_{h},{\dot{e}}_{h},{\ddot{e}}_{h},e_{h}^{\left(3\right)}\right]}^{T}$, *B*_*V*_ = [0,0, 1]^*T*^, and *B*_*h*_ = [0,0, 0,1]^*T*^. *ρ*_*V*_ and *ρ*_*h*_ denote, respectively, the designed nonpositive functions to improve the transient performance; *ξ*_*pV*_ and *ξ*_*ph*_ are the basis functions of the network; *ϖ* indicates its node parameter; ${\hat{w}}_{pV}$ and ${\hat{w}}_{ph}$ are the weights of the network; and *P*_*V*_ and *P*_*h*_ represent the positive definite solutions to the following Lyapunov equations:

(32) QV=−AVTPV+PVAV,Qh=−AhTPh+PhAh,

where *Q*_*V*_ and *Q*_*h*_ are selected as unit matrices, whereas

(33) AV=010001−kVp−kVd1−kVd2,Ah=010000100001−khp−khd1−khd2−khd3.

In addition, the update laws of the weights ${\hat{w}}_{pV}$ and ${\hat{w}}_{ph}$ are adopted as [21]

(34) w^˙pV=−γVEVTPVBVξpV,w^˙ph=−γhEhTPhBhξph,

where *γ*_*V*_ and *γ*_*h*_ represent the positive numbers, respectively. By applying the neural network outputs to compensate uncertain errors, the steady tracking performance will be ameliorated and the system robustness will be enhanced accordingly [22].

**Remark 1 .**

Let ${\hat{v}}^{*}$ be the best approximation with respect to *ε* where *ρ* = [*ρ*_*V*_, *ρ*_*h*_], *E* = [*E*_*V*_, *E*_*h*_], *P* = [*P*_*V*_, *P*_*h*_], *B* = [*B*_*V*_, *B*_*h*_], and the error bound is defined as

(35) $$\begin{array}{c} \left\|\;\varepsilon -{\hat{v}}^{*}\;\right\|\leq \tau . \end{array}$$

Also, the errors between $\hat{v}={\hat{w}}_{p}^{T}\xi_{p}$ and ${\hat{v}}^{*}$ are provided as

(36) v^−v^∗=w~pTξp,

(37) w~p=w^p−w^p∗,

where ${\hat{w}}_{p}^{*}$ is the weight with regard to ${\hat{v}}^{*}$, $\hat{v}=[{\hat{v}}_{pV},{\hat{v}}_{ph}{]}^{T}$, ${\hat{w}}_{p}=[{\hat{w}}_{pV},{\hat{w}}_{ph}{]}^{T}$, and *ξ*_*p*_ = [*ξ*_*pV*_, *ξ*_*ph*_]^*T*^. After substituting (31) and (36) into (30), we have

(38) $$\begin{array}{c} \dot{E}=AE+B\rho E^{T}PB+B{\tilde{w}}_{p}^{T}\xi_{p}+B\left(\;{\hat{v}}^{*}-\varepsilon \;\right), \end{array}$$

where *A* = [*A*_*V*_, *A*_*h*_], *P* = [*P*_*V*_, *P*_*h*_]. Furthermore, the Lyapunov function is defined as

(39) $$\begin{array}{c} L=\frac{1}{2}E^{T}PE+\frac{{\tilde{w}}_{pV}^{T}{\tilde{w}}_{pV}}{2\gamma_{V}}+\frac{{\tilde{w}}_{ph}^{T}{\tilde{w}}_{ph}}{2\gamma_{h}}. \end{array}$$

After taking the derivative with respect to (39), we obtain [18]

(40) L˙=−12ETQE+ETρPBBTPE+ETPBv^ad∗−ε≤−12E2+λ−ρPBBTPE2+τETPB≤−12E2+τETPB≤−ETPE2λ−P+τETPEλ−P.

Equation (40) is negative with

(41) $$\begin{array}{c} \sqrt{E^{T}PE}>2\tau {\left[\;\overset{-}{\lambda }\left(\;P\;\right)\;\right]}^{3/2}. \end{array}$$

Therefore, when *τ* → 0, then lim_*t*→0_⁡*E* → 0. On this basis, the nonlinear model becomes inaccurate when the airplane deviates from the design point, as a result of the fact that the inversion controller may be ineffective due to the unknown model information. In this case, the compensation output based on the neural network can cancel the model uncertainty and disturbance effect depending on the online adjustment of the weights, and the nonlinear portion can improve the transient performance, thus ameliorating global stability and self-adaptability for the overall system.

**Remark 2 .**

These functions, *ρ*_*V*_ and *ρ*_*h*_, change from 0 to the large negative numbers as the tracking error approaches zero [23]. At the initial condition, when controlled outputs *e*_*V*_ and *e*_*h*_ are far from the step commands, *ρ*_*V*_ and *ρ*_*h*_ are small because the influence of these nonlinear portions is constrained. In turn, when the track errors *e*_*V*_ and *e*_*h*_ reach the anticipated commands, in this case the nonlinear portion will become effective. In other words, *ρ*_*V*_ and *ρ*_*h*_ can guarantee large damping ratios of the closed system as controlled outputs reach the desired commands. To this end, the overshoot of the output response concerning the aircraft model will be reduced accordingly.

In general, the flight control law using *ρ*_*V*_ and *ρ*_*h*_ can achieve fast rising time for large tracking errors first. Once the system output approaches the step command, high damping ratio is set to remove the overshoot [24]. This achieves the following: not only can the flight velocity and altitude asymptotically track the step reference, but also the resulting closed-loop system can achieve better tracking performances and stronger robustness than those with the control law designed without the neural network and nonlinear part.

**Figure 1:**
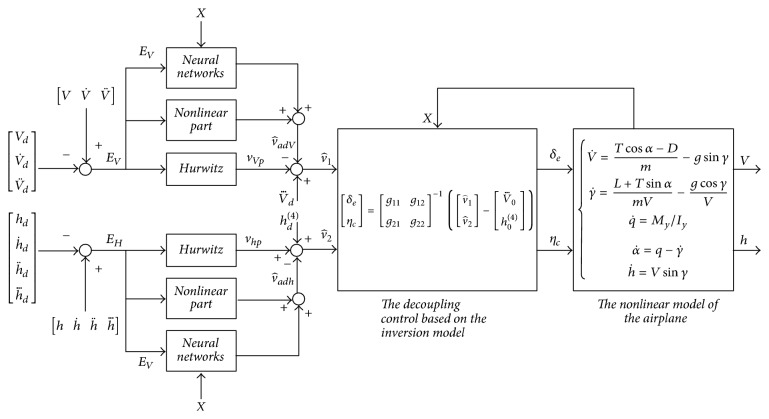
Structure diagram of robust adaptive control for the airplane.

In particular, the structure diagram of this robust adaptive control system is shown in Figure 1.

Figure 1 shows us that the designed control system includes the inversion control with Hurwitz, the nonlinear portion, and the adaptive compensation of neural networks. Among them, the inversion control is used to relieve the coupling dynamics of the nonlinear model, the nonlinear portion improves the transient performance, and the neural network output is applied to improve system robustness and adaptability. In brief, this proposed control law can not only provide the satisfactory control performance, but also ensure the system robustness over the overall flight envelope.

## 5. Illustrative Example

In this study, the airplane properties are used in [22]. The required aerodynamic coefficients and propulsive parameters are adopted based on [13], as well. Also, the relations between the acceleration of gravity and air density corresponding to the altitude are approximately provided by

(42) ρ=ρ0e−h/h0,g=g0R0R0+h2,

where *ρ*_0_ = 1.2266 kg/m^3^, *h*_0_ = 73152, *g*_0_ = 9.8 m/s^2^, and *R*_0_ = 6356766 m. Furthermore, we select the flight range as *V*∈ [4500 m/s, 4700 m/s] and *h*∈ [33500 m, 34000 m]. According to (5), the resulting trim states can be obtained. These states alter with the change in the different altitude and velocity in order to satisfy the balance condition. In addition, any small perturbation will lead to the divergence of the flight states in relation to the unstable and nonminimum phase dynamics [25], and designing a suitable control law is critical to ensure system stability and to relieve the coupling effects of the nonlinear model dynamics [26].

**Figure 2:**
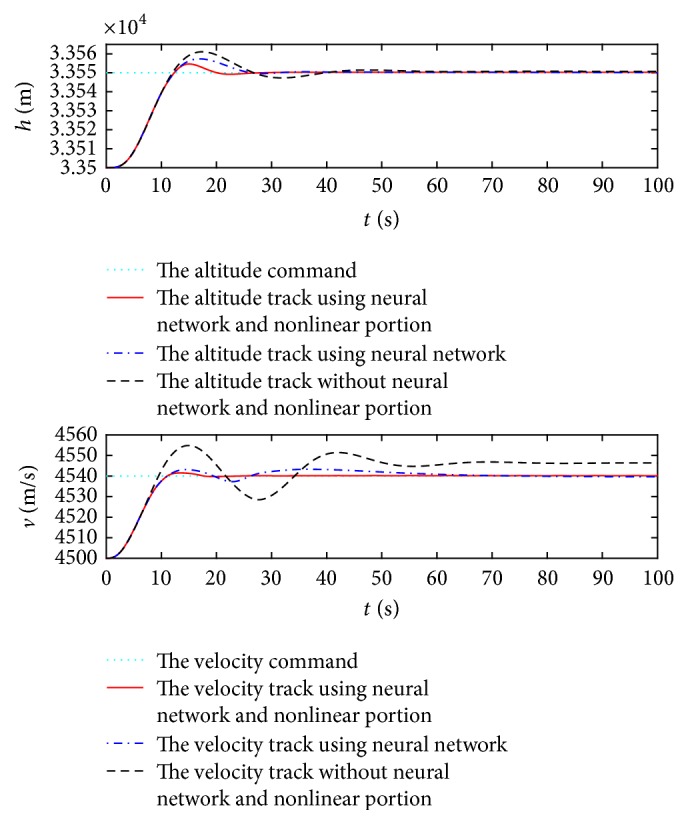
Response curves with regard to command signals.

First, the control goal is that the speed *V* and altitude *h* can follow rapidly the desired step commands Δ*V*_*c*_ = 40 m/s and Δ*h*_*c*_ = 50 m from the trimmed condition, respectively. During the response process at the first 200 seconds, the track results using the proposed controller are displayed in Figure 2.

Figure 2 shows that the track response without the neural network and nonlinear portion is undesirable, including the large track error, slow response time, and unintended overshoot, whereas the track qualities with only the neural network compensation can be improved, but the transient performance is not good. In comparison, when using the controller with the neural network and nonlinear portion, the velocity and altitude converge to the desired commands after 30 seconds, and this indicates that the proposed control law guarantees the decoupling and tracking performances of the closed system, and simultaneously the transient performance is satisfactory, including small steady-state error, rapid response time, and less overshoot.

**Figure 3:**
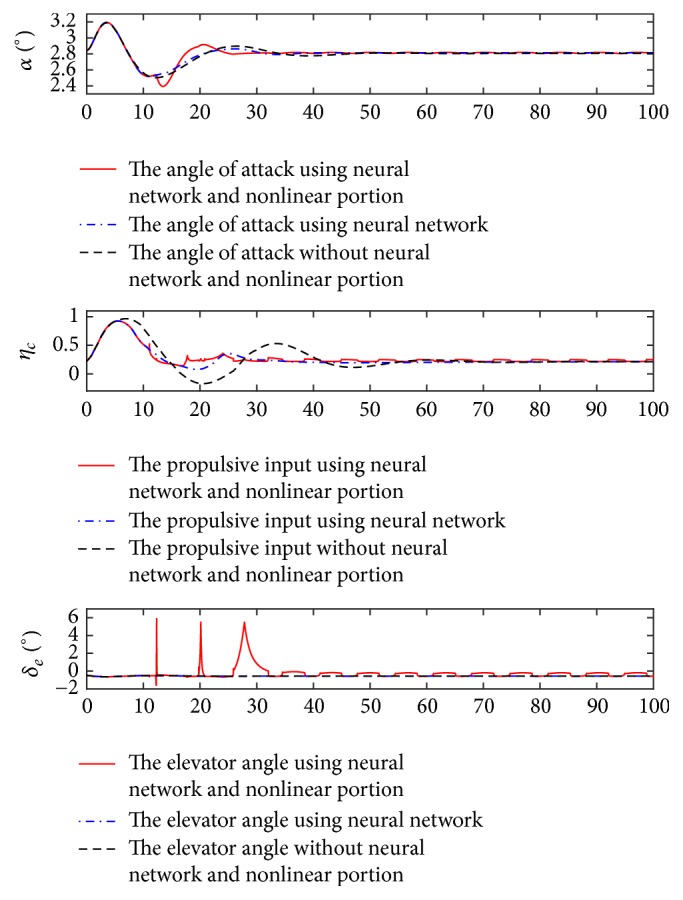
Change curves of angle of attack and control inputs.

Furthermore, the change curves corresponding to the angle of attack and control inputs are demonstrated in Figure 3. 

**Figure 4:**
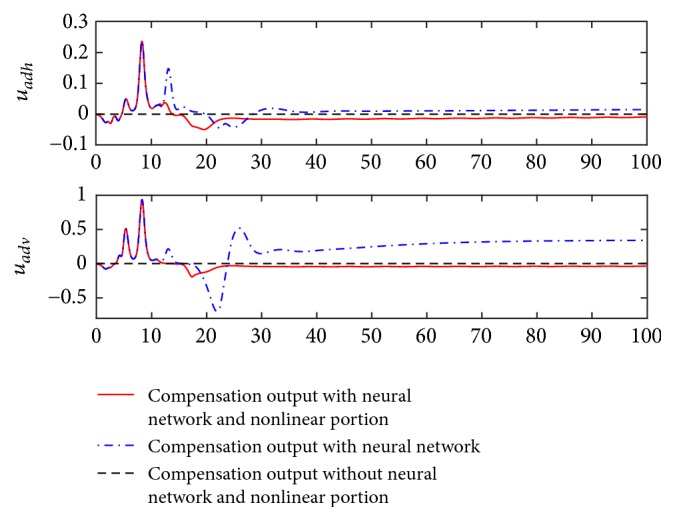
Compensation outputs.

From Figure 3, the angle of attack changes from the initial trim value to the anticipated value rapidly, and the elevator deflection and throttle setting vary smoothly and reasonably when the controller with the neural network and nonlinear portion is applied. These results illustrate the effectiveness of the presented controller to realize the decoupling control goal. Also, the compensation outputs of the neural network are demonstrated in Figure 4.

Figure 4 shows us that the compensation outputs of the neural network change adaptively with the system output, thus improving the accuracy concerning the inverse control. Particularly, as long as the track response reaches the new trim state, the resulting compensation signals will converge to the steady values such that flight stability can be guaranteed accordingly.

Furthermore, we assume that the model parameter matrix *G* in (14) is uncertain. In particular, the model parameters may change 20% or more due to the engine-airframe coupling action, mass loss, and sensor noise. To this end, we further consider the 20% random uncertainties of model parameters, and the proposed controller can solve the tracking control problem and tolerate the larger plant uncertainty. Thus, it is expressed by

**Figure 5:**
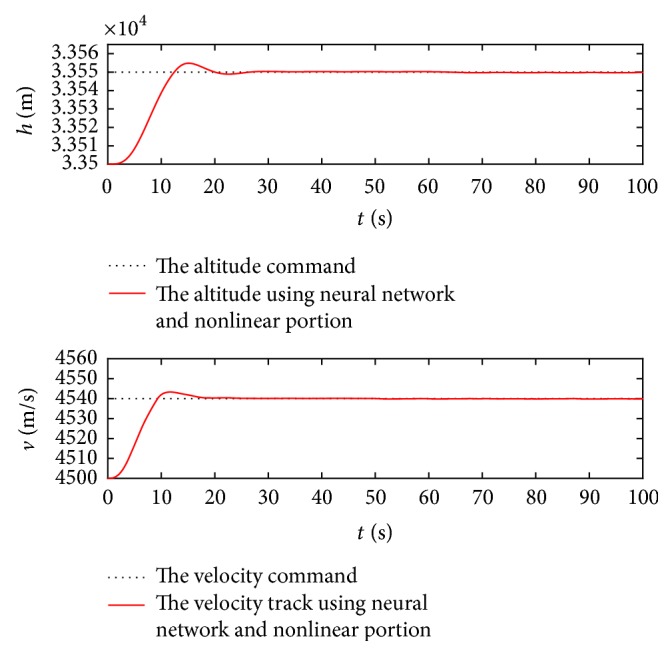
Response curves using adaptive control law in the uncertain condition.

**Figure 6:**
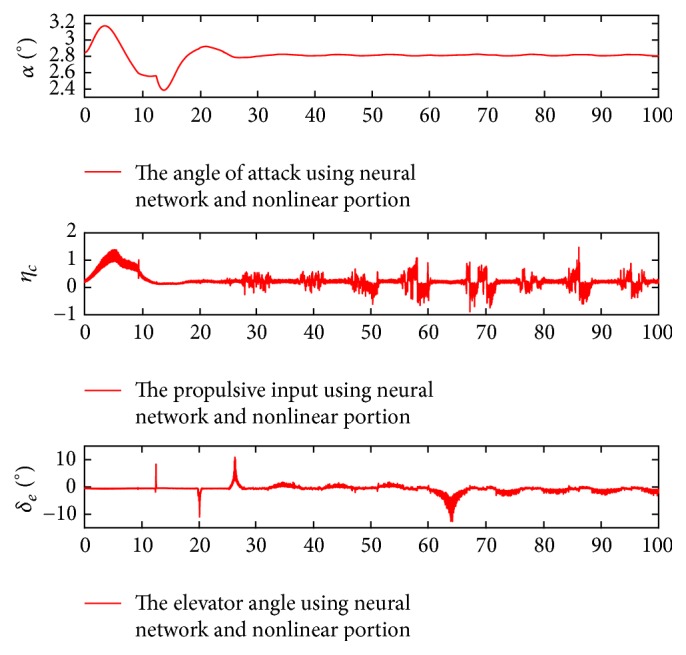
Change curves of angle of attack and control inputs in the uncertain condition.

(43) g11∗=g111+Δ11=ω13QfδemIy1+Δ11,g12∗=g121+Δ12=ω14Bm1+Δ12,g21∗=g211+Δ21=π13Dcos⁡γ+ω13Dmsin⁡γ1+Δ21,g22∗=g211+Δ22=π14Bcos⁡γ+ω14Bmsin⁡γ1+Δ22,

where Δ_11_, Δ_12_, Δ_21_, Δ_22_ are the uncertain items resulting from the sensor noise, modeling errors, inaccurate aerodynamic parameters, and so on. At this time, the presented control law with the neural network and nonlinear portion is adopted, and the simulation results accordingly can be exhibited in Figures 5 and 6.

Figures 5 and 6 show that the altitude and velocity follow the command signals quickly even in the large uncertain condition. Such results explain that the proposed controller can suppress the uncertain disturbances and guarantee system stability.

## 6. Conclusion

This paper proposes a control law using the neural-based inversion design approach with the nonlinear compensation for a general longitudinal model of the airplane. First, the nonlinear model of the airplane is established, and the balance equation is gotten for the given altitude and velocity. Next, the inversion control law is designed based on the feedback linearization principle. Furthermore, the control law in combination with the neural network and nonlinear portion is proposed. For this controller, the inversion control can realize the decoupling operation concerning the nonlinear model dynamics, whereas the adaptive outputs of the neural network and nonlinear portion can improve system robustness, transient performance, and adaptability. Finally, the simulation is conducted to show that the proposed control methods are feasible for a general longitudinal model of the airplane.
